# An Improved Method for Completely Uncertain Biological Network Alignment

**DOI:** 10.1155/2015/253854

**Published:** 2015-04-27

**Authors:** Bin Shen, Muwei Zhao, Wei Zhong, Jieyue He

**Affiliations:** ^1^School of Computer Science and Engineering, MOE Key Laboratory of Computer Network and Information Integration, Southeast University, Nanjing 210096, China; ^2^Division of Math and Computer Science, University of South Carolina Upstate, Spartanburg, SC 29303, USA

## Abstract

With the continuous development of biological experiment technology, more and more data related to uncertain biological networks needs to be analyzed. However, most of current alignment methods are designed for the deterministic biological network. Only a few can solve the probabilistic network alignment problem. However, these approaches only use the part of probabilistic data in the original networks allowing only one of the two networks to be probabilistic. To overcome the weakness of current approaches, an improved method called completely probabilistic biological network comparison alignment (C_PBNA) is proposed in this paper. This new method is designed for complete probabilistic biological network alignment based on probabilistic biological network alignment (PBNA) in order to take full advantage of the uncertain information of biological network. The degree of consistency (agreement) indicates that C_PBNA can find the results neglected by PBNA algorithm. Furthermore, the GO consistency (GOC) and global network alignment score (GNAS) have been selected as evaluation criteria, and all of them proved that C_PBNA can obtain more biologically significant results than those of PBNA algorithm.

## 1. Introduction

The development of biological experiment technology has generated more and more biological network data such as protein-protein interaction and gene transcriptional regulatory network data, which brings considerable number of pieces of information about the interactions and relationships between biological organisms. For this reason scientists carried out a lot of research in this area. Comparative analysis, namely, biological network alignment, is an important method in biological network research. Biological networks alignment can simply be described as the analysis of biological networks by comparing the data to find the correlation between structure and function of organisms and thus to help the study of biological development and evolution. This study demonstrates great potentials to discover basic functions and to reveal essential mechanisms for various biological phenomena, by understanding biological systems not at individual component level but at a system-wide level [1, 2].

Ogata et al. first proposed the graph comparison approach to identify local similarities between two graphs, which allows gaps and mismatch of nodes and edges and is especially suitable for detecting biological features in 2000 [3]. They used the above-mentioned comparative method to discover the relationship between enzymes and positions of their corresponding gene encodings in the entire genome. After analyzing these results, they found the local structure similarities corresponding to functionally related enzyme clusters. Thereafter, the graph comparison research attracted many scholars' research interests in this field. Kelley et al. in 2003 introduced the value of the concept called BLASTE into protein interaction network and thereby described a new way to detect the highly conserved pathway and the highly conserved functional module in the two networks [4]. Subsequently, Koyutürk et al. took advantage of the duplication/divergence model to translate protein-protein interaction (PPI) network comparison into the maximum weight subgraphs problems and used the greedy method to solve the problem [5]. In 2007, Singh et al. proposed the IsoRank algorithm, which converted the problem to a constraint-based optimization objective function problem. Then they also introduced an algorithm called IsoRankN, which was an approach similar to the PageRank-Nibble algorithm, to align multiple PPI network [6]. The Match-and-Split algorithm proposed by Narayanan and Karp, 2007, with the idea of dividing and conquering strategy divided biological networks into submodules. This approach deals with biological networks alignment by comparing smaller modules [7]. In 2009, Klau [8] normalized the problem to an optimization problem and solved the problem with the integer linear programming method. So far, most of the researches are focused on determining biological network data.

However due to the size, density, and redundancy of interacting molecules in the network and even errors in biological experiments [9], interactions in biological networks are probabilistic events. For example, in a living cell, DNA binding proteins are believed to be in equilibrium between the bound and unbound states, thus introducing uncertainties in protein-DNA interactions. Similar circumstance holds for protein-protein interactions, which are crucial to cellular functions both in assembling protein machinery and in signaling cascades. Therefore we abstract the biological networks into the uncertain networks whose edges are denoted by the values, respectively. Our solution is closer to realistic situation. Incorporation of uncertain information will bring more challenges to the biological networks alignment and analysis.

To the best of our knowledge, there are only two biological network alignment algorithms that can deal with uncertain network. Weighted IsoRankN [10] based on the IsoRank was proposed to deal with the probabilistic case. But the probability information in Weighted IsoRankN was considered as “weight" rather than the true “probability." Essentially, the Weighted IsoRankN algorithm merely simplifies the probability graph into the deterministic diagram. Hence a majority of pieces of information were neglected via this measure. PBNA (probabilistic biological network alignment) [11] proposed by Todor et al. in 2013 was an advanced version of the IsoRank algorithm. The core of this algorithm was to replace the determining variables in the IsoRank with a random variable so as to establish a model for biological network alignment problem. Then this probability algorithm was optimized by using conditional probability distribution knowledge to reduce its complexity. However, the PBNA approach requires at least one of the networks participating in alignment be determined diagram. In other words, if the participating networks are all uncertain networks, one of them will be considered automatically as a deterministic graph. Clearly, the neglect of the probability information of the networks may lead to the deviation.

In order to simplify the discussion in the rest of the cases, “deterministic network alignment” refers to the algorithm in which two participators are all deterministic network and “part probabilistic network alignment” refers to the algorithm in which one of participators is probability graph and “complete probabilistic network alignment” refers to the algorithm in which both participators are uncertain graphs.

In this paper, we develop a method called “complete probabilistic biological network alignment” (C_PBNA) based on “part probabilistic biological network alignment (PBNA).” Our approach can take full advantage of the information for the uncertain network alignment with two uncertain networks. Finally, we conduct 122 groups of contrast tests based on uncertain protein interactions network data preprocessed by Todor et al. from MINT database. We use the agreement to tell the difference between two algorithms. The biological significance of the network comparison is quantified by global network alignment score, gene ontology consistency, and functional coherence of the alignments. The experiment results indicate that C_PBNA can find the results neglected by PBNA algorithm. Furthermore, C_PBNA can obtain more biologically significant results than those of PBNA algorithm.

The rest of the paper is organized as follows. In Section 2, we describe the C_PBNA algorithm. In Section 3, we apply the C_PBNA algorithm into MINT [12] and analyze the results. In Section 4, the conclusions are given.

## 2. Methods

The C_PBNA proposed in the paper is an advanced biological networks alignment algorithm derived from the PBNA [9]. Both of the algorithms are based on the framework of IsoRank for deterministic network. The PBNA approach takes the uncertainties of the networks into consideration. However, the precondition of PBNA is that at least one of the biological networks participating in alignment must be deterministic network. Our approach can deal with alignment of two uncertain biological networks. Furthermore it can directly deal with the deterministic and partially probabilistic situation. In the following sections, we start by analyzing the probabilistic biological networks which are dealt with by the C_PBNA algorithm then and the results discovered by C_PBNA. Our whole approach is described in five sections: (1) Probabilistic Biological Network; (2) Probabilistic Support Matrix; (3) Probabilistic Model of the Eigenvector; (4) Extracting Alignment Results; (5) C_PBNA Algorithm.

### 2.1. Probabilistic Biological Network

Traditional deterministic biological network can be characterized by a two-tuple *G* = (*V*, *E*), where *V* denotes the vertex and *E* denotes the set of graph edges. Different types of biological networks correspond to different graphs; for instance, PPI network (protein-protein interaction network) can be abstracted into an undirected graph with the vertices tagged by labels denoting different proteins and the edges denoting the interaction relationship of proteins.

It is important to note that biological networks usually are indeterminate; for instance, the interactions of proteins often exist at certain probability. Therefore, we consider the uncertain biological network as a network in which the proteins are denoted by determinate nodes and the interactions of proteins are denoted by edges with a probability value.

Uncertain network is characterized by a three-tuple *g* = (*V*, *E*, *Pr*⁡_*E*_), where *V*, *E* denote the vertex set and edge set, respectively [13]. Consider

(1) $$\begin{array}{c} {\mathrm{Pr}}_{E}:E\to \left[0,1\right]. \end{array}$$

Note that *Pr*⁡_*E*_ is a function which denotes a value probability in [0,1] for each edge *e* = (*u*, *v*); specifically *Pr*⁡_*e*_ = 1 indicates that edge *e* = (*u*, *v*) definitely exists:

(2) Pr⁡g⟹G =∏e∈E′Pr⁡Ee∏e∈E∩(V′×V′)∖E′1−Pr⁡Ee.

*g* = (*V*, *E*, *Pr*⁡_*E*_) denotes uncertain graph and *G* = (*V*′, *E*′) denotes deterministic graph, respectively. Particularly, uncertain graph *g* contains deterministic *G*, which is abbreviated as *g*⇒*G*, when and only when *V*′ = *V* and *E*′⊆*E*∩(*V*′ × *V*′), where *E*∩(*V*′ × *V*′) denotes an edge set in which two endpoints of the edge are both in the vertex set *V*′.

**Example 1 .**

**Figure 1:**
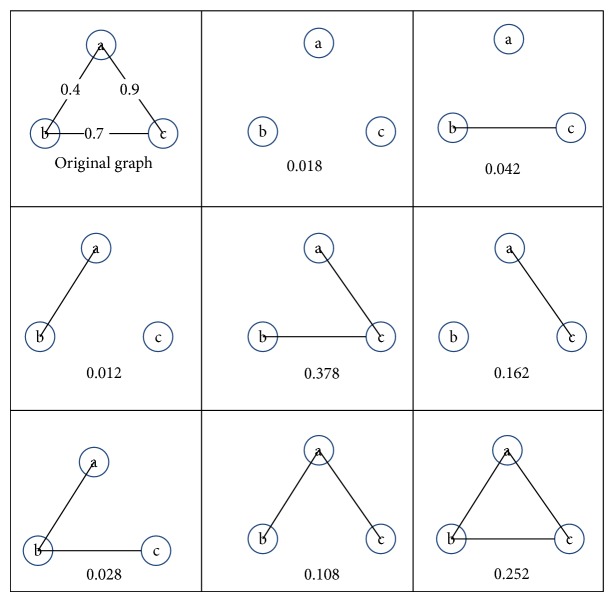
Original uncertain graph, as well as its eight-implication graph.

Observe that original uncertain graph in Figure 1 has three probability sides. Hence, it contains 8 kinds of different deterministic networks with different probability. This means that in a probabilistic network with |*E*| edges there are actually 2^|*E*|^ deterministic networks which occur at a certain probability.

Note that, with the uncertainty added, the complexity of the graph increases greatly. For instance, the MINT [12] network data used in the experiments contain 2^313^ implication graphs for the maximum organism containing 313 edges. Precise comparison seems almost impossible for such a large amount of data.

### 2.2. Probabilistic Support Matrix

Firstly, our approach proposed in the paper is based on the primal framework of IsoRank algorithm which aimed at deterministic graph alignment. One of the core ideas of IsoRank is that the similarity between two vertices may be determined by all the neighborhood vertices' similarity. First of all, we introduce the simple case of pairwise global network alignment (GNA).

For deterministic networks *G*_1_ = (*V*_1_, *E*_1_) and *G*_2_ = (*V*_2_, *E*_2_), the similarity score *R*_*ij*_ between vertexes *v*_*i*_ and *v*_*j*_ can be calculated by

(3) $$\begin{array}{c} R_{ij}=\underset{u\in N\left(i\right)}{\sum }\;\underset{v\in N(j)}{\sum }\frac{1}{d_{u}d_{v}}R_{uv}, \end{array}$$

where *v*_*i*_ ∈ *V*_1_, *v*_*j*_ ∈ *V*_2_, *N*(*i*), *N*(*j*), respectively, denote the neighbor vertexes set of *v*_*i*_ and *v*_*j*_, and *d*_*u*_, *d*_*v*_, respectively, denote the degrees of *v*_*u*_ and *v*_*v*_. We assume that *m* = |*V*_1_|, *n* = |*V*_2_|, all similarity scores *R*_*ij*_  (0 ≤ *i* ≤ *m*, 0 ≤ *j* ≤ *n*) constituting an *m* × *n* dimensional similarity score vector *R*. *R* can be seen as a vector form converted from an *m* × *n* matrix. Therefore (4) can be rewritten in matrix form: *R* = *AR*, where

(4) $$\begin{array}{c} A\left[i,j\right]\left[u,v\right]=\left\{\begin{array}{cc} \frac{1}{d_{u}d_{v}}, & \text{if}\;\left(v_{i},v_{u}\right)\in E_{1},\;\left(v_{j},v_{v}\right)\in E_{2}\\ \frac{1}{mn}, & \text{if}\;d_{u}d_{v}=0\\ 0, & \text{otherwise}, \end{array}\right. \end{array}$$

where *A*[*i*, *j*][*u*, *v*] is a (*mn*)×(*mn*) matrix with double indexed row and column. And *A*[*i*, *j*][*u*, *v*] denotes the element of [*i*, *j*] rows and [*u*, *v*] column of the matrix *A*. The term 1/*mn* denotes that point *d*_*u*_ or point *d*_*v*_ is an acnode. As can be seen, formula, *R* = *AR*, indicates that vector *R* is a characteristic feature vector of matrix *A* when the eigenvalue is 1.

The important improvement of PBNA algorithm is to replace the deterministic variable in original algorithm with a random variable so as to simplify the model by calculating the expectation *E*(*A*) instead of *A* itself. It should be stressed that, due to the complexity in calculating desired *E*(*A*), PBNA alignment algorithm requires that one of the graphs must be determined. Considering this idea as a reference, we propose C_PBNA algorithm which can be extended to the network alignment problem in which two graphs, *G*_1_ and *G*_2_, are both uncertain graphs. Hence, the degrees of uncertain graph nodes set of *v*_*u*_ and *v*_*v*_ are both uncertain rather than deterministic values. The degree values *d*_*v*_, *d*_*u*_ are denoted by discrete random variables *D*_*v*_, *D*_*u*_ respectively, and then (4) can be rewritten as

(5) $$\begin{array}{c} A\left[i,j\right]\left[u,v\right]=\left\{\begin{array}{cc} \frac{1}{D_{u}D_{v}}, & \text{if}\;\left(v_{i},v_{u}\right)\in E_{1},\;\left(v_{j},v_{v}\right)\in E_{2}\\ \frac{1}{mn}, & \text{if}\;D_{u}D_{v}=0\\ 0, & \text{otherwise}, \end{array}\right. \end{array}$$

where *D*_*v*_, *D*_*u*_ are discrete distribution: *P*(*D*_*v*_ = *k*), *k* = 0,1,…, *d* ^max⁡^, *D*_*v*_ is the degree distribution of node *v*_*v*_. We assume that *E*_*v*_ is a set of edges connecting to point *v*_*v*_; hence, *P*(*D*_*v*_) can be obtained via probabilistic graphical models shown in Table 1.

Clearly, adding uncertain information increases the complexity of the algorithm. As a result, the time complexity for calculating each node degree distribution sequence increase to exponential time, because the neighbor points degrees in the matrix *A* as an item subject to the discrete distribution rather than a certain value. Therefore, based on the core idea of literature [9], we use *E*(*A*) instead of *A* involved in the calculation. The following section summarizes the calculation arriving at expectation *E*(*A*) of matrix *A*.

### 2.3. Probabilistic Model of the Eigenvector

We start by discussing (5) in the first case. Clearly, as discussed earlier, (*v*_*i*_, *v*_*u*_) ∈ *E*_1_, (*v*_*j*_, *v*_*v*_) ∈ *E*_2_; hence *v*_*u*_ and *v*_*v*_ have at least one connecting edge. So bring *D*_*u*_ = *k*_1_  (*k*_1_ = 1,…, *d*_*u*_^max⁡^), *D*_*v*_ = *k*_2_, (*k*_2_ = 1,…, *d*_*v*_^max⁡^) into (5) as follows:

(6) EAi,ju,v=∑k1∈Du,k2∈Dv1k1k2 ·PAi,ju,v=1k1k2 ∣ vi,vu∈E1,vj,vv∈E2.

Because of assuming that the edges of the network *G*_1_ and *G*_2_ are independent events, so the *D*_*u*_ and *D*_*v*_ are independent too, we can derive as follows:

(7) EAi,ju,v=∑k1∈Du,k2∈Dv1k1k2·PDu=k1 ∣ vi,vu∈E1·PDv=k2 ∣ vj,vv∈E2.

In the next case of (5), the probability is denoted by *P*(*D*_*u*_ *D*_*v*_ = 0). Note that *D*_*u*_ and *D*_*v*_ are also independent; we can get (8), after some manipulations as follows: *E*[*A*[*i*, *j*][*u*, *v*]] = (1/*mn*)*P*(*D*_*u*_ *D*_*v*_ = 0)

(8) $$\begin{array}{c} E\left[A\left[i,j\right]\left[u,v\right]\right]=\frac{1}{mn}\left(P\left(D_{u}=0\right)+P\left(D_{v}=0\right)\right). \end{array}$$

Similarly, substituting the results of (7) and (8) for *P*_0_ and *P*_*k*_1_*k*_2__, respectively, we obtain

(9) $$\begin{array}{c} E\left(A\left[i,j\right]\left[u,v\right]\right)=\frac{1}{mn}\times P_{0}+\overset{d_{u}^{\max}}{\underset{k_{1}=1}{\sum }}\;\overset{d_{v}^{\max}}{\underset{k_{2}=1}{\sum }}\frac{1}{k_{1}k_{2}}\cdot P_{k_{1}k_{2}}, \end{array}$$

where the probability distributions of *D*_*u*_ and *D*_*v*_ are calculated as discussed in Table 1; thus we get the *E*(*A*). However, as discussed above in Section 2.2, calculating *P*(*D*_*u*_ = *k*) directly means that the computational complexity of the algorithm can reach *O*(2^*d*_*v*_+*d*_*u*_^). In order to reduce the high complexity, we use the probability generating function introduced in the literature.

**Definition 2 (see [14]).**

Assume that *X* is a discrete random integer variable ranging from 0 to *N*; therefore the probability generating function (PGF) of *X* may be defined as a polynomial of *z*:

(10) $$\begin{array}{c} Q_{X}\left(z\right)=E\left[z^{X}\right]=\overset{N}{\underset{k=0}{\sum }}P\left(X=k\right)z^{k}. \end{array}$$

As we see in Definition 2, the coefficient distribution sequence corresponds to discrete random variables distribution of *X* in probability generating function. Clearly, as long as the probability generating function is calculated, the probability distribution will be obtained easily. Moreover, the probability generating function may be calculated by Theorem 3.

**Theorem 3 (see [14]).**

Suppose that *G* = (*V*, *E*, *Pr*⁡_*E*_) is an uncertain graph and *E*_*v*_ denotes a set of edges connecting with *v* endpoint; hence, the degree of *v* is a discrete random variable whose probability generating function is shown as

(11) $$\begin{array}{c} Q_{N_{v}}\left(z\right)=\underset{e\in E_{v}}{\prod }\left(1-p_{e}+p_{e}z\right). \end{array}$$

For example, Figure 1 shows an uncertain network, in which there are two edges connecting with the node and the appearance probability of those two edges is 0.4 and 0.9, respectively. As a result, the probability generating function of degree distribution for node *a* is *Q*_*N*_*a*__(*z*) = (0.6 + 0.4*z*)(0.1 + 0.9*z*) = 0.06 + 0.58*z* + 0.36*z* ^2^; then we can easily get the distribution of degree for node *a* as in Table 2.

Therefore, we can calculate the probability generating function of the node degree distribution for *v* and then obtain node distribution sequence via the probability generating function. The computational complexity of this process can decrease Naive Approach Complexity from *O*(2^*d*_*v*_^max⁡^+*d*_*u*_^max⁡^^) to *O*((*d*_*v*_^max⁡^ *d*_*u*_^max⁡^)^2^).

Our ultimate objective is the conditional probability distribution of the degree. In other words, the purpose is to calculate the distribution sequence of node *v* with the presupposition that there exists an edge connecting to node *v* and edge *e* ∈ *E*_*v*_. As a result, the probability generating function of the conditional probability can be obtained by simply dividing (1 − *p*_*e*_ + *p*_*e*_ *z*).

Now we can easily calculate the probability generating function of the conditional probability *P*(*D*_*v*_ = *k*∣*e* ∈ *E*_*v*_)  *k* = 1,2,…, *d*_*v*_^max⁡^. And we can get the support matrix *E*(*A*) within the time expected in the polynomial equation (9). In particular, the sequence similarity method is also added to the score vector according to the literature [5] as

(12) $$\begin{array}{c} R=\alpha E\left(A\right)R+\left(1-\alpha \right)E, \end{array}$$

where *E* is the vector denoting normalization of sequence similarity score BLAST and *α* is a constant used for balancing influence of topological similarity and sequence similarity on calculating the pairwise similarity. Finally, we use a power iteration method [15, 16] to calculate *R* and record all similarity score. See Algorithm 2.

### 2.4. Extracting Alignment Results

After calculating similarity vector *R*, the last step of our model is to extract the final alignment results from vector *R*. In order to extract the final alignment results, we introduce a breadth first searching approach by using maximum weight bipartite matching technique [17, 18]. First of all, we introduce the concept of bipartite graph and maximum weight bipartite matching.

Bipartite graph: a graph *G*_12_ = (*V*_12_, *E*_12_) is bipartite if there exists *V*_12_ = *V*_1_ ∪ *V*_2_ with *V*_1_∩*V*_2_ = *∅*. And for each edge *e* ∈ *E*_12_, the two end vertices must belong to the two different subsets *V*_1_ and *V*_2_.

Maximum weight bipartite matching: given a bipartite graph *G*_12_ = (*V*_12_, *E*_12_) with bipartition (*V*_1_, *V*_2_) and weight function *w* : *E* → *R* find matching of maximum weight where the weight of matching *M* is given by *w*(*M*) = ∑_*e*∈*E*_12__ *w*(*e*).

The process of extracting results from the feature vector *R* is the process to find a max-weight matching *M* in *G*_12_.

Let us call a function *y* : (*V*_1_ ∪ *V*_2_) → *R* a potential if *y*(*i*) + *y*(*j*) ≤ *w*(*i*, *j*) for each *i* ∈ *V*_1_, *j* ∈ *V*_2_. The value of potential is ∑_*v*∈*V*_1_∪*V*_2__ *y*(*v*). It can be seen that the cost of each perfect matching is at least the value of each potential. The Hungarian method finds perfect matching and a potential with equal value which proves the optimality of both. In fact it finds perfect matching of tight edges: an edge *e*_*ij*_ is called tight for a potential if *y*(*i*) + *y*(*j*) = *w*(*i*, *j*). See Algorithm 3.

### 2.5. C_PBNA Algorithm

**Figure 2:**
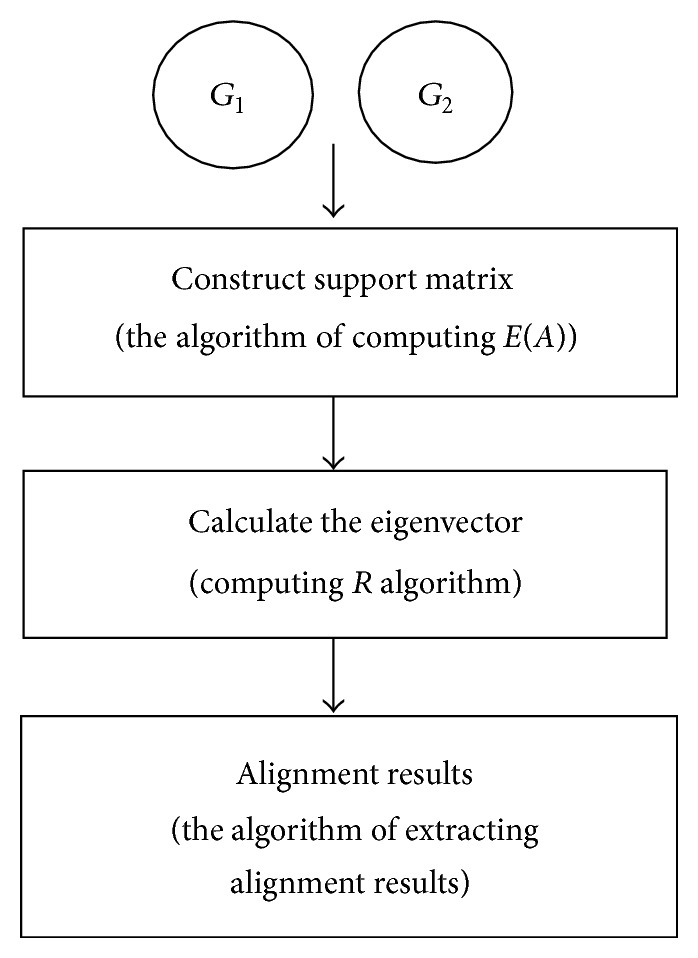
The framework of C_PBNA.

The C_PBNA algorithm can be roughly divided into three steps, constructing the support matrix, calculating the eigenvector of the matrix, and extracting alignment results, as in Figure 2. These steps will give detailed descriptions by Algorithms 1, 2, and 3.

First we build probabilistic support matrix based on the conclusions of Section 2.2 and calculate *E*(*A*) based on formula (9) in Section 2.3. The pseudocode can be seen in Algorithm 1. Secondly, we calculate the feature vector *R* by using an iterative approach denoted as in Algorithm 2. Thirdly, we extract optimal comparison by interpreting *R* as encoding a bipartite graph and finding the maximum weight bipartite matching, which is denoted as in Algorithm 3.

In Algorithm 1 we have the following.

1. *Line 1–Line 4.* Construct the PGF for every node in probabilistic networks *G*_1_ and *G*_2_.
2. *Line 5–Line 14*. Calculate the probability *P*_0_ corresponding to the values of 1/*mn*.
3. *Line 15–Line 21*. Calculate the 1/*k*_1_ *k*_2_ of probability *P*_*k*_1_*k*_2__ corresponding to *Q*_*D*_*u*__^*i*^‖_*k*−1_ which represents *k* − 1 coefficient of the probability generating function *Q*_*D*_*u*__^*i*^.

After getting the desired *E*(*A*), we use an iterative approach to calculate the feature vector *R*. Set each of values *R*_*ij*_ in the eigenvalue *R* equal to constant 1/*mn*, the original variable *R* called *R*_0_. *E* indicates the normalized vector of sequence homologies. The *α* values have been studied in the literature [5, 16], so we directly use the best value 0.6. *ε* is a sufficiently small constant; iteration will eventually converge to approximate similarity score vector *R*. The process calculation of *R* is shown in Algorithm 2.

In Algorithm 2 we have the following.

1. *Line 1–Line 4*. Set the initial value of the feature vector *R*_0_.
2. *Line 5–Line 9*. There is iterative calculation until the two values of feature vector difference are less than the set value of  *ε*.

After getting the feature vector *R*, the last step of the C_PBNA algorithm is extracted by final comparison results from *R* as shown in Algorithm 3. C_PBNA adopted the method mentioned in Section 2.4; this method is to find perfect matching *M*.

In Algorithm 3 we have the following.

1. *Line 1–Line 3*. Build bipartite graph *G*_12_ and compute the weight matrix by the feature vector *R*.
2. *Line 3–Line 5*. Set the initial value of the *y*, *E*_12_, *M*.
3. *Line 7–Line 11*. Find an optimal augmenting path cover (*v*_*u*_, *v*_*v*_) by the max-flow min-cut theorem and then update the feasible labeling *y*.
4. *Line 12–Line 14*. Update *E*_12_, *M* until *M* is perfect matching.

## 3. Experiments and Results

The experiments in this research include two main parts. The first part shows that C_PBNA algorithm can obtain the results which are neglected by PBNA. Further, the second part of the experiments proves that results of C_PBNA are more biologically significant using GOC and GNAS (global network alignment score) as evaluation standards.

Experimental environment described in Table 3 indicates the conditions of conducting the experiment designed in this study. Besides, we make use of QT (a cross-platform application framework) library function directly to deal with problems associated with array, matrix, and sorting in PBNA and C_PBNA algorithm. The QT library function is available at http://qt-project.org/. In addition, the OGDF library function which can be obtained at http://ogdf.net/ is used to read and query biological network data.

The uncertain dataset used in the experiment obtained from the MINT database is network data of protein-protein interactions preprocessed by Todor et al. [9, 10]. As a result, providing that MINT network is of enough biological importance, the network information offered by KEGG database is divided into several smaller networks. Then, only the network with more than 10 nodes remains. Finally, we obtain 198 protein-protein interaction networks coming from 10 organisms.  Table 4 shows statistical information of this network.

There are 198 networks comparing with each other, which will result in *C*_198_^2^ = 19503 groups of experiments. However the networks through KEGG are divided into a set of vertices function associated with proteins, and KEGG used a label to mark the set of proteins. The proteins from different sets share less similarity, which makes little sense to do the network comparison. Therefore we can get 122 groups of comparison experiments from the KEGG database.

### 3.1. Coherence Comparison of C_PBNA and PBNA

In order to prove that C_PBNA can discover the results neglected by PBNA, agreement evaluation criterion [9] is introduced in this research.

The definition of agreement is based on the same dataset. Hence, the proportion of common results discovered by both C_PBNA and PBNA in all alignments is shown as

(13) $$\begin{array}{c} \frac{\left|\text{Alignments}\;\text{in}\;\text{common}\right|}{\left|\text{All}\;\text{alignments}\right|}. \end{array}$$

The score of this evaluation criterion is between 0 and 1. The larger the score is, the more common results these two algorithms have. Particularly, it shows that the results of both methods agree perfectly if the evaluation criterion equals 1 while it means that the results of these two algorithms are completely different if the evaluation criterion equals 0.

**Figure 3:**
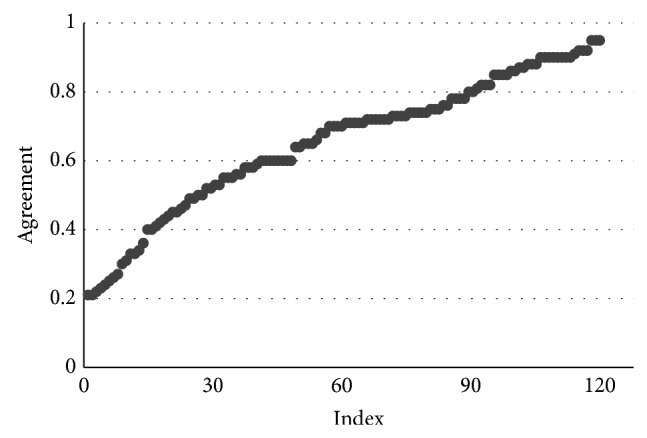
Agreement.

In this research, 122 groups of experiments are conducted and the result agreements of C_PBNA and PBNA algorithms are figured out in each group of experiment. Finally, we get 122 agreements plotted by the number of experiments on the horizontal axis and agreements on the vertical axis as shown in Figure 3.

The ordinate values are the 122 agreement values after sorting, and the abscissa values are the serial number of the experiment.

Table 5 shows the detailed agreement statistics of 122 groups of experimentation. In particular, the left Pie Chart is divided into 4 parts corresponding to the percentage of each category and Category 1 is not described in the Pie Chart due to its percentage of 0. For instance, Category 5 in Table 5 indicates that there are 30 experiments with the agreement between 0.8 and 1, which accounts for 24.6% of the total experiments.

**Figure 4:**
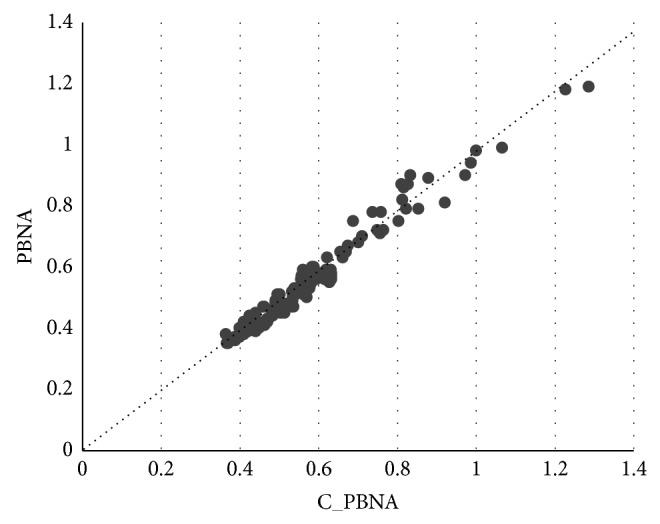
GOC statistics of PBNA and C_PBNA.

The general distribution of the agreements is shown intuitively in Figure 4. We can see that the Agreement values are distributed within the range from 0.2 to 0.95. From the Pie Chart, we can further see that agreement scores less than 0.8 experiments accounted for 75% of the experiments, among which only 14% of the total experiment is less than 0.4 points. It indicates that both the C_PBNA results and PBNA results have many overlapping parts but have noticeable difference at the same time. The reason is that one of the most basic differences is that C_PBNA concludes all of the uncertain information while the PBNA method only utilizes half the uncertain information.

Therefore, we may draw a conclusion that neglecting uncertain information could lead to deviation. In addition, all the above shows that much more innovative result can be obtained through C_PBNA algorithm. The corresponding biological significance will be demonstrated by the following experiments.

### 3.2. Gene Ontology Consistency Comparisons of C_PBNA and PBNA

Gene ontology consistency (GOC) has been generally used to measure the biological significance of alignment results and we use it to evaluate biological meaning of alignment result by

(14) $$\begin{array}{c} \text{GOC}=\underset{\langle u,v\rangle \in V_{12}}{\sum }\frac{\left|\text{GO}\left(u\right)\cap \text{GO}\left(v\right)\right|}{\left|\text{GO}\left(u\right)\cup \text{GO}\left(v\right)\right|}, \end{array}$$

where GO(*u*) denotes the set of GO terms which label a protein *u* in gene ontology database.

Then, the GOC of each pair of proteins in alignment results is calculated, respectively. The bigger the GOC is, the more similar function these proteins have; especially, the maximum of GOC is 1 which means that these proteins have totally the same function. All GO data in this study comes from GO Consortium [19] and literature [14].

Similarly, in order to get alignment results of C_PBNA and PBNA, respectively, the GOC (the value of GOC is between 0 and 1) is calculated for each pair of proteins in 122 groups of experiments. Finally, we get 122 groups of results, among which there are 2 GOCs in each group. The distribution of the GOC is shown in Figure 4 and Table 6.

In Figure 4, *x*-coordinate denotes GOC value of C_PBNA algorithm and *y*-coordinate denotes GOC value of PBNA algorithm. Table 6 shows 122 groups of GOC value and the diversity of every two GOC values in each group.

As we can see in Table 6, for most of the results the value of *x*-coordinate is larger than *y*-coordinate. For instance, it includes 96 groups of experiments, 78.7% of the total experiments, in Category 3 and Category 4. Furthermore, the *x*-coordinate is higher than the *y*-coordinate more than 10% in 17 groups of experiment. These all indicate that in most of experiments C_PBNA algorithm may discover more biologically significant results based on the same evaluation standard GOC.

In conclusion, C_PBNA and PBNA can obtain diverse results for biological networks alignment which is proved through the first experiment. Moreover, C_PBNA is demonstrated in the second experiment to be superior to PBNA in discovering biologically significant results since it uses all uncertain information while the PBNA algorithm neglects some uncertain information in biological networks.

### 3.3. Functional Coherence of the Alignments

The functional coherence of the alignments is motivated by the lack of automated and direct measures of ortholog-list quality. Comparing with GOC, the functional coherence of the alignments reports the average of the medians instead of the sum. And it maps each GO term to one or more of a standardized set of GO terms.

**Figure 5:**
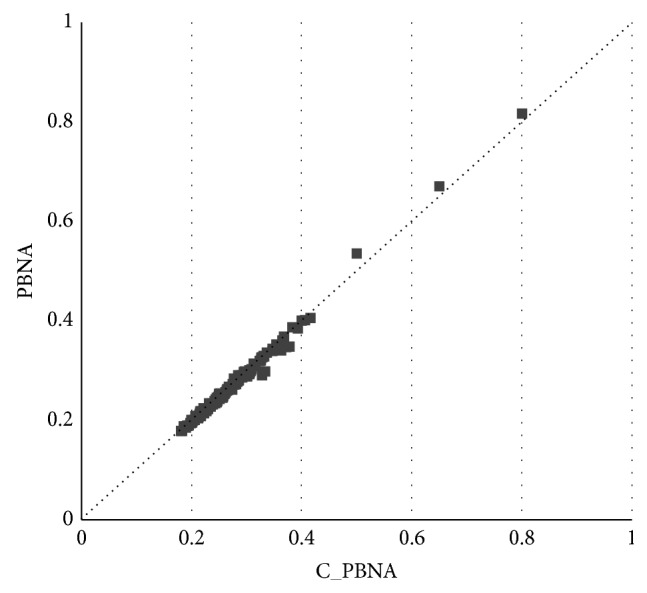
Functional coherence of alignments using PBNA and our method C_PBNA.

We can see in Figure 5 that PBNA and C_PBNA have very similar functional coherence values with only a few minor differences. One of the reasons is that the functional coherence function computes the similarity of a standardized set of GO terms instead of the aligned proteins directly. The other reason is that it reports the average of the medians, so it cannot tell whether a mapping has many highly similar terms or not. Since the median of a distribution is not an accurate representation of the entire distribution, the result it returns is not sensitive enough to tell the difference between different alignments.

### 3.4. GNAS Valuation Comparison of C_PBNA and PBNA

As one of the biological networks alignment algorithms, GNAS (global network alignment score) [20] is adopted as evaluation criterion in this paper defined in (15). Specifically the larger value of GNAS indicates more conserved interactions and higher sequence similarity:

(15) $$\begin{array}{c} \text{GNAS}=\alpha \times \left|E\right|+\left(1-\alpha \right)\times \sum \text{seq}\left(u,v\right). \end{array}$$

**Figure 6:**
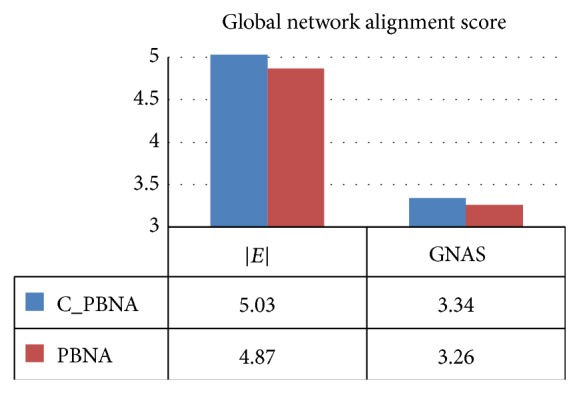
Comparison of |*E*| and GNAS from C_PBNA and PBNA.

In this formula, seq(*u*, *v*) denotes sequence similarity values of the two nodes; |*E*| denotes the number of edges of *G*_12_ (for the definition of *G*_12_, please refer to Section 2.4). Based on the common dataset, two groups of GNAS with 122 values in each group are obtained through C_PBNA and PBNA, respectively. The average values of GNAS and |*E*| are showed in Figure 6.

As we can see in Figure 6, the values of |*E*| and GNAS obtained from C_PBNA are superior to those from PBNA since C_PBNA adopts full uncertain information which increases the amount of conserved interactions.

### 3.5. Time Analysis

The running time of PBNA and C_PBNA is evaluated in this experiment. The most time-consuming step for both algorithms is constructing similarity matrix, which takes about 90% of the entire running time. Therefore, it is reasonable that we measure only this step's running time in order to evaluate the entire algorithm time efficiency. The results are shown in Table 7.

Table 7 indicates that the time spent in constructing similarity in C_PBNA is longer than its counterpart in PBNA, because C_PBNA deals with both probabilistic networks, which takes more information into consideration. Network comparison with two uncertain networks is much more complex as we can see in Sections 2.1 and 2.2. When computing *E*(*A*), in fact, the complexity of C_PBNA is *O*((*d*_*v*_^max⁡^ *d*_*u*_^max⁡^)^2^) while the complexity of PBNA is *O*((*d*_*v*_^max⁡^)^2^) by PGF method mentioned above in Section 2.3. Although C_PBNA spends more time dealing with both probabilistic networks, its time performance is still acceptable. Furthermore, the results which we get have more biological significance, and C_PBNA can deal with the probabilistic networks directly instead of the preprocessing and transforming of the data into deterministic network.

## 4. Conclusions

Biological networks alignment is an important topic in bioinformatics. However, the network data has its inherent complexity of and the combine optimizes features of biological networks alignment are not clear, which make relevant algorithm study extremely challenging. A majority of the classic biological networks alignment algorithms are based on deterministic network while the alignment method for probabilistic networks is still under discussion.

In this paper, we propose a complete probabilistic model and a complete probabilistic biological algorithm for network comparison. Our approach has several advantages. First, our approach is based on complete probabilistic network, which takes the uncertainties of both networks instead of the single one into consideration. Consequently, our approach can take full advantage of the uncertainties properties of network comparison. Second, we model the network alignment using two probability matrices. Therefore, the uncertainties can be quantified by the probabilities of connections in the networks. As a result, our approach is capable of comparing two networks which both have uncertain properties. Third, we use a unified probabilistic model for different types of network alignment (deterministic, part probabilistic, and complete probabilistic), unlike other alignments which use different methods for different types of networks. Finally, the evaluation criteria including GOC and GNAS are used in the experiments to demonstrate that the results of C_PBNA and PBNA are different and that the results of the former algorithm are more biologically significant.

Usually, affinity propagation in probabilistic networks is random and probability factors have not been taken into consideration in this paper, and the effect of these factors on results will remain an open problem for the future research. The computational time increases as a result of using more probability information, which is a subject we will study in the next step.

## Figures and Tables

**Algorithm 1:**
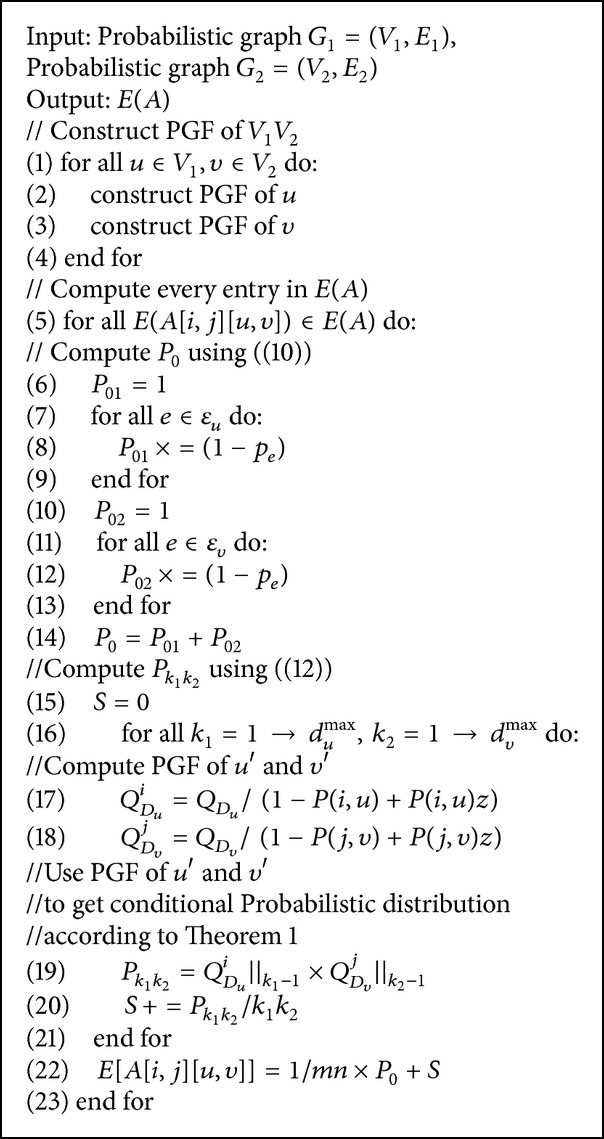
The algorithm of computing E(A).

**Algorithm 2:**
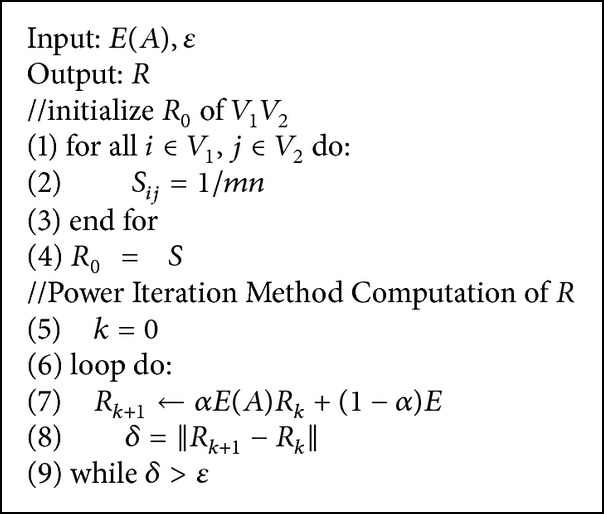
Computing *R* algorithm.

**Algorithm 3:**
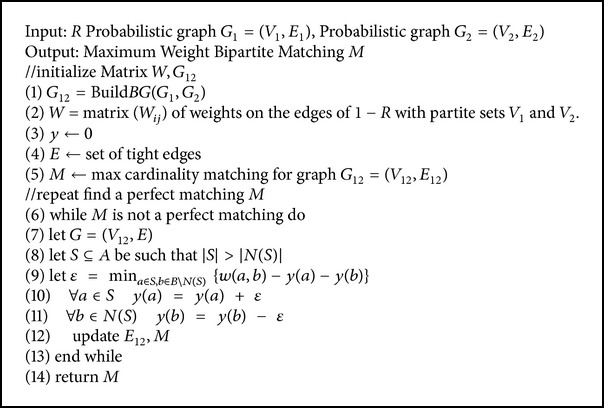
Extracting alignment results.

**Table 1:** Degree distribution of nodes.

*D*	*P*(*D*)
0	$\prod_{e\in E}\left({1-\mathrm{Pr}}_{E}\left(e\right)\right)$

1	$\underset{e_{i}\in E}{\sum }{\mathrm{Pr}}_{E}\left(e_{i}\right)\prod_{e\in E/e_{i}}\left({1-\mathrm{Pr}}_{E}\left(e\right)\right)$

⋮	⋮

*d* ^max⁡^	$\prod_{e\in E}{\mathrm{Pr}}_{E}\left(e\right)$

**Table 2:** Degree distribution of node *a*.

*N*_*a*_	0	1	2

P(*N*_*a*_)	0.06	0.58	0.36

**Table 3:** Experimental environment.

Experimental environment	

Programming environment	QT, C++

Library function	QT and OGDF library function

Hardware environment	CPU clock speed of 3.3 GHz, memory of 4 G

**Table 4:** Experimental data.

Organism	Number of networks	Number of proteins		Number of interactions	
		Average	Max	Average	Max
Cel	7	14.00	22	9.57	21
Dme	7	17.14	28	12.42	26
Eco	6	16.83	27	21.16	26
Hpy	1	11.00	11	7.00	7
Has	83	36.50	96	46.55	168
Mmu	43	16.23	40	11.16	33
Rno	13	14.69	30	11.00	22
Sce	34	32.91	106	80.32	313
Spo	3	11.00	11	10.00	10
Tpa	1	20.00	20	21.00	21

**Table 5:** Agreement statistics.

Category	Agreement	Quantity	Percentage
1	0–0.2	0	0%
2	0.2–0.4	16	13.1%
3	0.4–0.6	33	27.0%
4	0.6–0.8	43	35.2%
5	0.8–1	30	24.6%

Total	0–1	122	100%

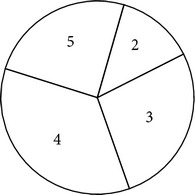			

**Table 6:** GOC statistical data.

Category	Diversity	Quantity	Percentage
1	<−10%	0	0%
2	−10%–0%	26	21.3%
3	0%–10%	79	64.8%
4	>10%	17	13.9%

Total	−∞–+∞	122	100%

**Table 7:** PBNA and C_PBNA algorithm time statistics.

Method	Average (second)	Max (second)
PBNA	125.4	545.5
C_PBNA	490.1	9014.6
